# The Dipyridamole Added to Dual Antiplatelet Therapy in Cerebral Infarction After First Acute Myocardial Infarction: A Nationwide, Case-Control Study

**DOI:** 10.3389/fneur.2018.01003

**Published:** 2018-11-27

**Authors:** Mei-Tzu Wang, Cheng Ken Tsai, Shu-Hung Kuo, Wei-Chun Huang, Kun-Chang Lin, Wang-Ting Hung, Chin-Chang Cheng, Pei-Ling Tang, Cheng Chung Hung, Jin-Shiou Yang, Hsin-Li Liang, Guang-Yuan Mar, Chun-Peng Liu

**Affiliations:** ^1^Department of Critical Care Medicine, Kaohsiung Veterans General Hospital, Kaohsiung, Taiwan; ^2^Department of Cardiovascular Surgery, Zuoying Branch of Kaohsiung Armed Forces General Hospital, Kaohsiung, Taiwan; ^3^National Defense Medical Center, National Defense University, Taipei, Taiwan; ^4^School of Medicine, National Yang-Ming University, Taipei, Taiwan; ^5^Department of Physical Therapy, Fooyin University, Kaohsiung, Taiwan; ^6^Graduate Institute of Clinical Medicine, Kaohsiung Medical University, Kaohsiung, Taiwan

**Keywords:** acute myocardial infarction, antiplatelet agent, dipyridamole, stroke, dual antiplatelet therapy

## Abstract

**Background and Purpose:** No previous study has compared the impact of dipyridamole-based triple antiplatelet therapy on secondary stroke prevention and long-term outcomes to that of dual antiplatelet therapy (DAPT) in patients with acute myocardial infarction (AMI) and previous stroke. This study aimed to evaluate the impact of dipyridamole added to DAPT on stroke prevention and long-term outcomes in patients with cerebral infarction after first AMI.

**Methods:** This nationwide, case-control study included 75,789 patients with cerebral infarction after first AMI. A 1:4 propensity score matching ratio was adopted based on multiple variables. Finally, the data of 4,468 patients included in the DAPT group and 1,117 patients included in the Dipyridamole-DAPT group were analyzed. Primary outcome was overall survival. Secondary outcomes were cumulative event rate of recurrent MI or stroke, and cumulative intracerebral hemorrhage (ICH) and gastrointestinal bleeding rate.

**Results:** Long-term survival rate was comparable between the two groups (log-rank *P* = 0.1117), regardless of sex analyses. However, after first year, DAPT subgroup revealed better survival over DAPT-dipyridamole subgroup (log-rank *P* = 0.0188). In age subgroup analysis, a lower survival rate was detected in younger patients from the Dipyridamole-DAPT group after first year (log-rank *P* = 0.0151), but no survival difference for older patients. No benefit of Dipyridamole-DAPT was detected for patients after AMI, regardless of the myocardial infarction type. DAPT was superior to Dipyridamole-DAPT in patients who underwent percutaneous coronary intervention (PCI) (log-rank *P* = 0.0153) and ST elevation myocardial infarction after first year (log-rank *P* = 0.0019). Dipyridamole-DAPT did not reduce cumulative event rate of recurrent MI or stroke in patients after AMI. Moreover, Dipyridamole-DAPT increased the cumulative ICH rate (log-rank *P* = 0.0026), but did not affect the cumulative event rate of gastrointestinal bleeding. In Cox analysis, dipyridamole did not improve long-term survival.

**Conclusions:** This nationwide study showed that Dipyridamole-DAPT, compared with DAPT, did not improve long-term survival in patients with stroke after AMI, and was related to poor outcomes after 1 year. Dipyridamole-DAPT did not reduce recurrent rate of MI or stroke, but increased the ICH rate without impacting the incidence of gastrointestinal bleeding.

## Introduction

Blockade of upstream activation or downstream aggregation of platelets alone cannot completely impede thrombus formation (1). Multiple antiplatelet agents acting via various pathways and employing complimentary mechanisms of action are necessary to effectively prevent thrombus formation (2). However, thrombotic events frequently occur after stenting in patients with acute myocardial infarction (AMI), despite the conventional dual antiplatelet (DAPT) treatment (3–5). To overcome this problem, different regimens of triple antiplatelet therapy (TAPT) were previously studied. Glycoprotein (GP) IIb/IIIa inhibitors are used as bailout therapy, and are limited to situations of angiographic evidence of a large thrombus, slow- or no-reflow phenomenon, and other thrombotic complications occurring during percutaneous coronary intervention (PCI) (6, 7). Cilostazol-based TAPT was found to reduce the risks of major adverse cardiac events (MACE) and all-cause mortality among patients with AMI after PCI, without increasing the risk of bleeding (8). Moreover, some studies have shown additional benefits of cilostazol-based TAPT for patients undergoing coronary stenting, especially with drug-eluting stent (DES) implantation, and long coronary lesions (9–12), but these benefits were not confirmed by other trials (13, 14).

For patients with high-grade arterial stenosis of atherosclerotic origin and at high-risk of stroke, early short-term DAPT (e.g., aspirin and clopidogrel) is effective in reducing the risk of recurrent stroke without increasing the risk of bleeding compared with antiplatelet monotherapy (15–17). The superiority of DAPT in these patients raised the hypothesis of the benefit of TAPT, providing the bleeding risk is not excessive. *In vitro*, the combination of aspirin, dipyridamole, and ARC69931 (a direct-acting antagonist of the ADP receptor, with a mechanism of action similar to that of clopidogrel) showed superiority to dual therapy or monotherapy in inhibiting platelet activation (1, 18). However, human studies failed to show the benefits of Dipyridamole-DAPT in the secondary prevention of stroke, but found a significantly increased risk of major bleeding associated with this therapy (19, 20).

Previous studies showed inconclusive results on the efficacy and safety of TAPT (cilostazol-DAPT) after AMI or stable angina for prevention of recurrent stroke and myocardial infarction (MI) (8–14). For patients with AMI and previous stroke, no previous study investigated the impact of Dipyridamole-DAPT on secondary stroke prevention and long-term outcomes compared with DAPT. The aim of this study was to evaluate the impact of dipyridamole added to DAPT on stroke prevention and long-term outcome in patients with cerebral infarction after first AMI.

## Methods

### Data sources

Taiwan launched a single-payer National Health Insurance program since 1 March 1995. This system has been providing universal health coverage and equal medical access to ~99% of Taiwan residents. Taiwan National Health Insurance Research Database (NHIRD) is a computerized database that includes data collected from more than 23 million patients. This database includes encrypted de-identification numbers, inpatient medical records on demographic information, International Classification of Diseases, Ninth Revision, Clinical Modification (ICD-9-CM) diagnostic codes, and drug codes.

This database has been extensively used in epidemiologic studies in Taiwan. This study collected the data from the NHIRD from January 2000 to December 2012. The Human Research Committee of Kaohsiung Veterans General Hospital approved this study.

### Definition of AMI population

**Figure 1:**
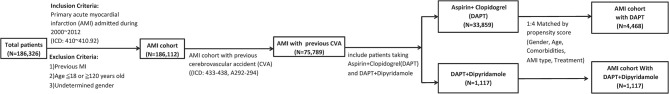
Flow-chart of the study cohort. There were 186,326 patients in Taiwan between January 2000 and December 2012 with a primary diagnosis of AMI (IC codes: 410–410.92). Patients were excluded who had a previous admission for AMI, were aged ≤ 18 or ≥120 years old, and whose sex was undetermined. Among the AMI cohort of 186,112 cases, 75,789 patients who were previously diagnosed with cerebrovascular accidents (CVA) (ICD: 433–438, A292–294) were analyzed. We included 1,117 patients taking aspirin and clopidogrel in DAPT group, and 33,859 patient taking DAPT plus dipyridamole in DAPT-dipyridamole group. Propensity score matching in a ratio of 1:4 based on variables of gender, age, hypertension, dyslipidemia, DM, PVD, ESRD, COPD, and PCI was adopted. Finally, we enrolled 4,468 patients in the DAPT group with aspirin and clopidogrel and 1,117 patients in the DAPT-dipyridamole group in our final analysis. AMI, acute myocardial infarction; CVA, cerebrovascular accident; DAPT, dual antiplatelet therapy; DM, diabetes mellitus; PVD, peripheral vascular disease; ESRD, end-stage renal disease; COPD, chronic obstructive pulmonary disease; PCI, percutaneous coronary intervention.

A total of 186,326 patients with a primary diagnosis of AMI (ICD: 410–410.92) were retrieved from NHIRD in Taiwan between January 2000 and December 2012 as the AMI cohort. Patients aged under 18 years or over 120 years, with a previous admissions for AMI, or with undetermined sex were excluded. Finally, 186,112 patients were included in the analysis (Figure 1).

### Study population

Among the AMI cohort of 186,112 cases, the data of 75,789 patients who were previously diagnosed with cerebrovascular accidents (CVA) (ICD: 433–438, A292–294) were analyzed. A total of 33,859 patients taking aspirin and clopidogrel were included in the DAPT group, and 1,117 patients taking DAPT plus dipyridamole were included in the DAPT-dipyridamole group. Propensity score matching at a ratio of 1:4 based on variables of sex, age, hypertension, dyslipidaemia, diabetes mellitus (DM), peripheral vascular disease (PVD), end-stage renal disease (ESRD), chronic obstructive pulmonary disease (COPD), and percutaneous coronary intervention (PCI) was adopted. Finally, 4,468 patients from the DAPT group receiving aspirin and clopidogrel, and 1,117 patients from the DAPT-dipyridamole group were included in the analysis (Figure 1).

### Outcome analysis

The measurement of mortality was based on the end date of the NHI coverage, with a maximum error of 1 month, because of monthly paid and easily canceled coverage of the NHI premium (21–23). Survival was defined based on the difference between the date of hospitalization and the end date of NHI coverage (24).

Primary outcome was overall survival. Secondary outcomes were cumulative event rate of recurrent MI or stroke, and cumulative intracerebral hemorrhage (ICH) and gastrointestinal bleeding rate. We defined “younger (age < 75)” and “older (age ≧ 75)” patients by using age of 75 as a cut point in light of CHA2DS2-VASc score (25).

### Statistical analysis

For data analysis, SAS software version 9.4 (SAS Institute Inc., Cary, NC) was used. All variables were calculated using descriptive statistics. Percentile values were used to express categorical data. Mean and standard deviation (SD) were used to report continuous data. The chi-squared test was used to analyse categorical data, while the paired *t*-test was used to compare continuous variables. The hazard ratio (HR) and associated 95% confidence intervals (95% CIs) were obtained from Cox proportional hazard regression models. Kaplan–Meier cumulative survival curves were used to compare the outcomes between the DAPT and DAPT-dipyridamole groups based on sex, age, ST elevation myocardial infarction (STEMI), non-ST elevation myocardial infarction (NSTEMI), DM, and PCI. *P* < 0.05 were considered statistically significant. To clarify and identified each delicacies of the beginning crossed over part of Kaplan–Meier survival curve, we analyzed the curve into “within 1 year” and “1–10 years” in overall, age, STEMI, and NSTEMI subgroups.

## Results

The descriptive characteristics of 4,468 patients from the DAPT group and 1,117 patients from the DAPT-dipyridamole group are listed in Table 1. No differences were found between the two groups in terms to age, sex, comorbidities, and PCI. Regarding medication, no differences were detected in the use of angiotensin-converting enzyme inhibitors (ACEIs) or angiotensin receptor blockers (ARBs), beta-blockers, statins, and nicorandil between the two groups. However, patients in the DAPT group received more frequently heparin or low molecular weight heparin (*P* = 0.002), while DAPT-dipyridamole subgroup was prescribed more nitrate (*P* = 0.0008) (Table 1).

**Table 1:** Characteristics of patients with previous cerebral infarction after first hospitalization for AMI in the subgroups of the dual antiplatelet therapy (DAPT) and Dipyridamole-DAPT groups (*N* = 5,585).

**Characteristics**	**ASA**+ **Clopidogrel (DAPT)**		**Dipyridamole-DAPT**		***P*-value**
	**(*****N*** = **4,468)**		**(*****N*** = **1,117)**		
Male	2,742	(61.37%)	687	(61.5%)	0.9343
Age ≧ 75	2,289	(51.23%)	575	(51.48%)	0.8829
**AMI TYPE**					
STEMI	1,051	(23.52%)	266	(23.81%)	
NSTEMI	3,417	(76.48%)	851	(76.19%)	0.8377
**COMORBIDITIES**					
Hypertension	3,459	(77.42%)	872	(78.07%)	0.6419
Dyslipidemia	2,343	(52.44%)	584	(52.28%)	0.9253
Diabetes mellitus	2,515	(56.29%)	630	(56.4%)	0.9462
Peripheral vascular disease	234	(5.24%)	64	(5.73%)	0.5125
End Stage renal disease	284	(6.36%)	73	(6.54%)	0.8269
Chronic obstructive pulmonary disease	771	(17.26%)	197	(17.64%)	0.7638
**TREATMENT**					
Percutaneous coronary intervention	1,924	(43.06%)	485	(43.42%)	0.8289
**DRUGS**			
ACEIs or ARBs	3,130	(70.05%)	766	(68.58%)	0.3364
Statins	1,816	(40.64%)	447	(40.02%)	0.7028
Beta blockers	2,578	(57.7%)	663	(59.36%)	0.3158
Heparin or LMWH	3,869	(86.59%)	927	(82.99%)	0.0020
Aldosterone antagonists	778	(17.41%)	221	(19.79%)	0.0642
Nitrates	3,833	(85.79%)	1,001	(89.62%)	0.0008
Nicorandil	519	(11.62%)	139	(12.44%)	0.4426

*ACEI, angiotensin-converting enzyme inhibitor; ARB, angiotensin receptor blocker; LMWH, low Molecular weight heparin; STEMI, ST elevation myocardial infarction; NSTEMI, non-ST elevation myocardial infarction*.

**Figure 2:**
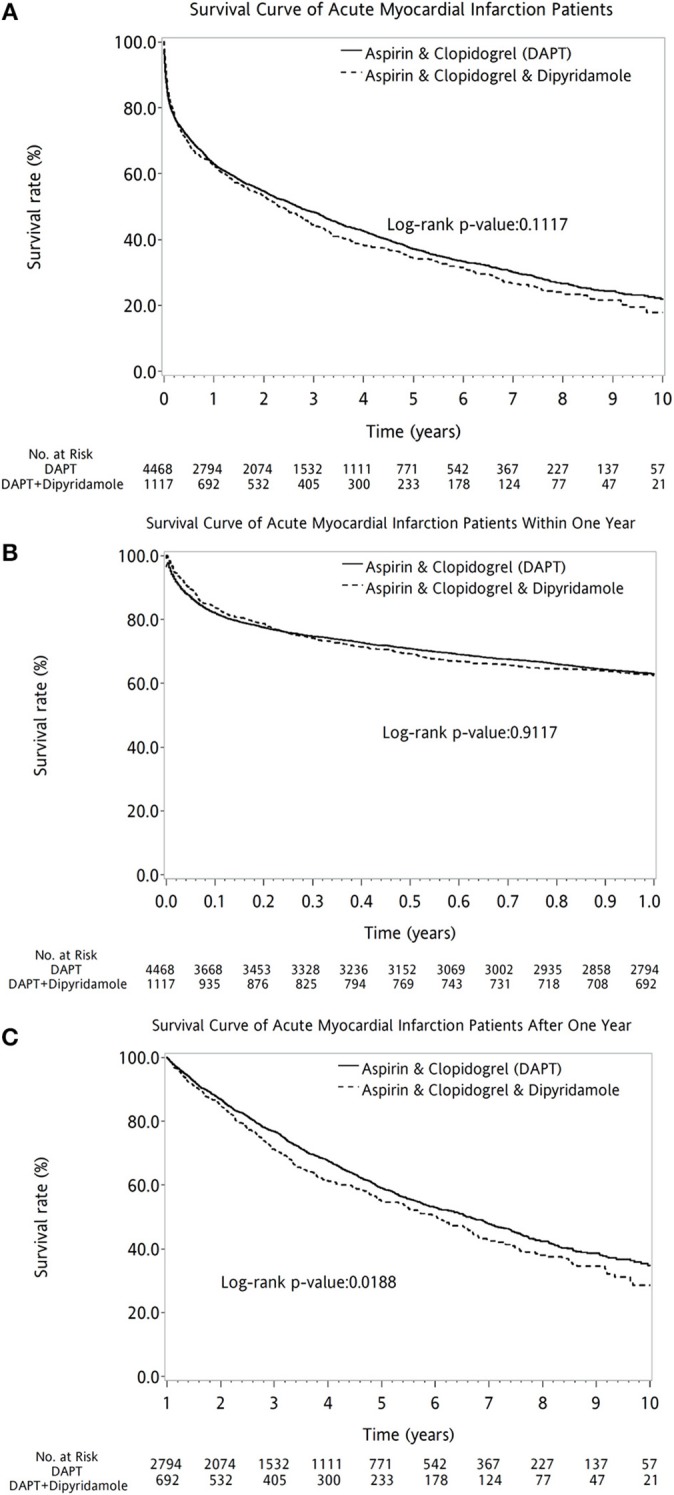
The comparison of long-term outcome between DAPT and Dipyridamole-DAPT groups in patients with previous stroke after first AMI, using Kaplan–Meier survival curve. Overall, the 10-year survival rate was comparable between the two groups of patients (log-rank *P* = 0.1117, **A**). Before the first year, overall survival rate was no difference between DAPT and Dipyridamole-DAPT subgroups (log-rank *P* = 0.9117, **B**). However, significant better survival of DAPT was shown after the first year with compare to Dipyridamole-DAPT (log-rank *P* = 0.0188, **C**). AMI, acute myocardial infarction; DAPT, dual antiplatelet therapy.

**Figure 3:**
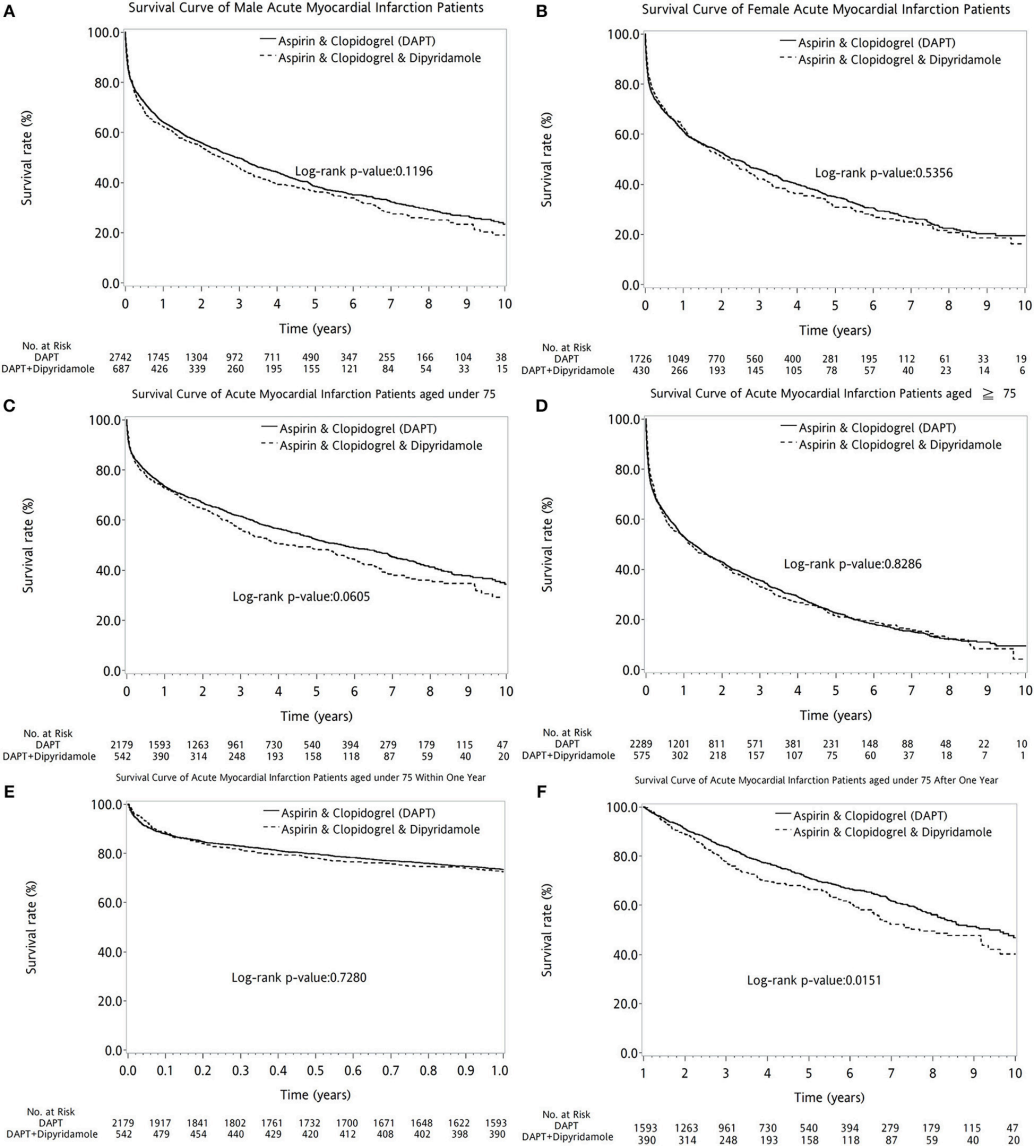
The comparison of long-term outcome between DAPT and DAPT-dipyridamole groups in patients with cerebral infarction after first acute myocardial infarciton (AMI) in sex and age subgroup analysis. Similar long-term outcomes were detected in men (log-rank *P* = 0.1196, **A**) and women (log-rank *P* = 0.5356, **B**). In age subgroup analysis, both younger (log-rank *P* = 0.0605, **C**) and older patients (log-rank *P* = 0.8286, **D**) showed comparable survival rate between DAPT and Dipyridamole-DAPT groups. However, in further subanalysis, DAPT had significant better overall outcome in younger patients after the first year (log-rank *P* = 0.0151, **F**). But, this benefit was not found within 1 year (log-rank *P* = 0.7280, **E**). AMI, acute myocardial infarction; DAPT, dual antiplatelet therapy.

Overall, the 10-year survival rate was comparable between the two groups of patients (log-rank *P* = 0.1117, Figure 2A). In further subanalysis, overall survival rate during first year was no difference between DAPT and DAPT-dipyridamole subgroups (log-rank *P* = 0.9117, Figure 2B). However, significant better overall outcome of DAPT was shown after the first year (log-rank *P* = 0.0188, Figure 2C). Whereas, Similar long-term outcomes were detected in men (log-rank *P* = 0.1196, Figure 3A) and women (log-rank *P* = 0.5356, Figure 3B). In age subgroup analysis, both younger (log-rank *P* = 0.0605, Figure 3C) and older patients (log-rank *P* = 0.8286, Figure 3D) showed comparable survival rate between DAPT and Dipyridamole-DAPT groups. However, in further subanalysis, DAPT had significant better overall outcome in younger patients after the first year (log-rank *P* = 0.0151, Figure 3F). But, this benefit was not found within 1 year (log-rank *P* = 0.7280, Figure 3E).

**Figure 4:**
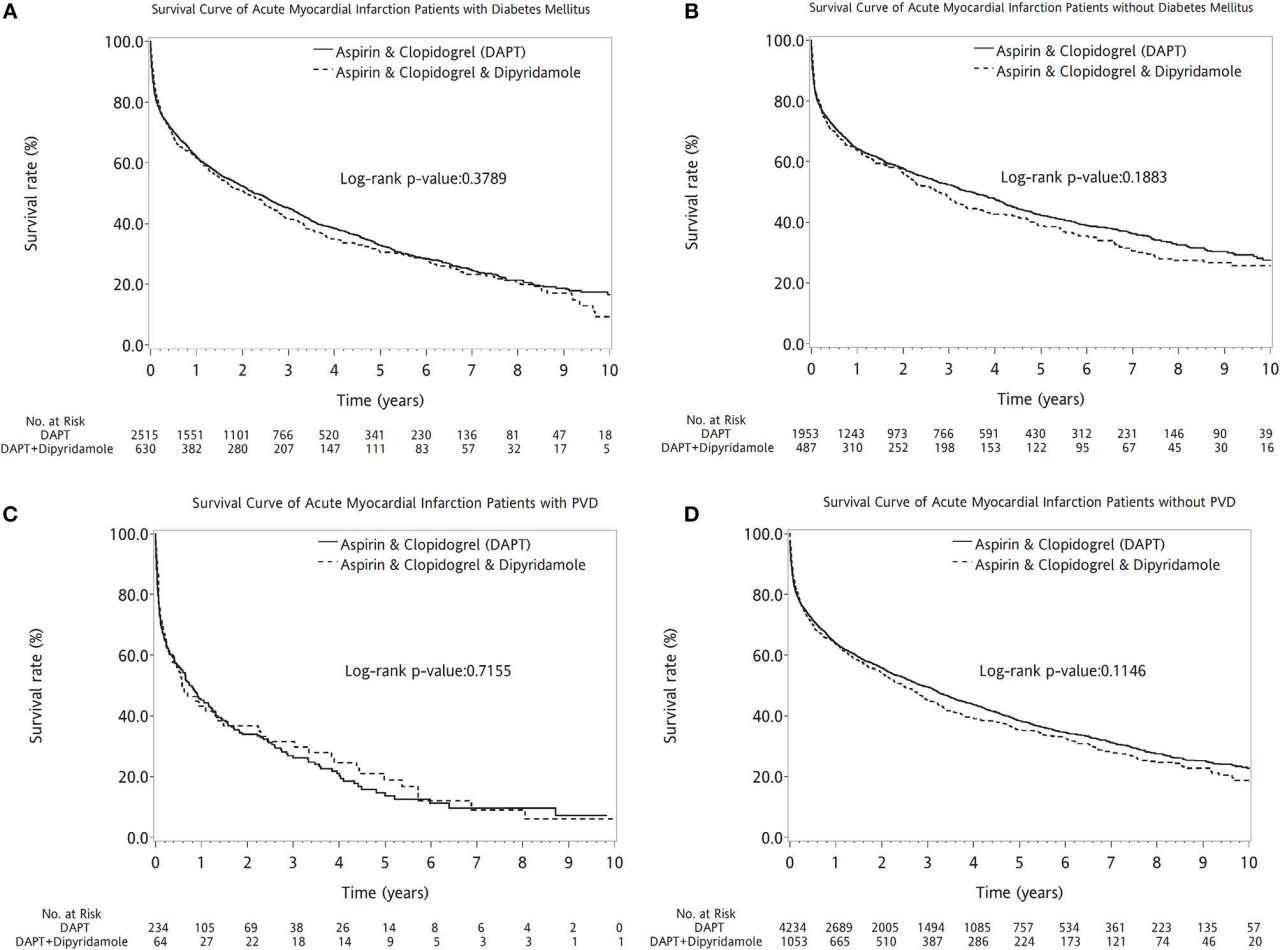
Kaplan–Meier survival curve in patients with previous stroke after first acute myocardial infarciton (AMI) in DM and PVD subgroup analysis. The two therapeutic strategies revealed no statistical differences between patients with (log-rank *P* = 0.3789, **A**) and without DM (log-rank *P* = 0.1883, **B**). No superiority of either therapy was detected between patients with (log-rank *P* = 0.7155, **C**) or without PVD (log-rank *P* = 0.1146, **D**). AMI, acute myocardial infarction**;** DM, diabetes mellitus; PVD, peripheral vascular disease.

**Figure 5:**
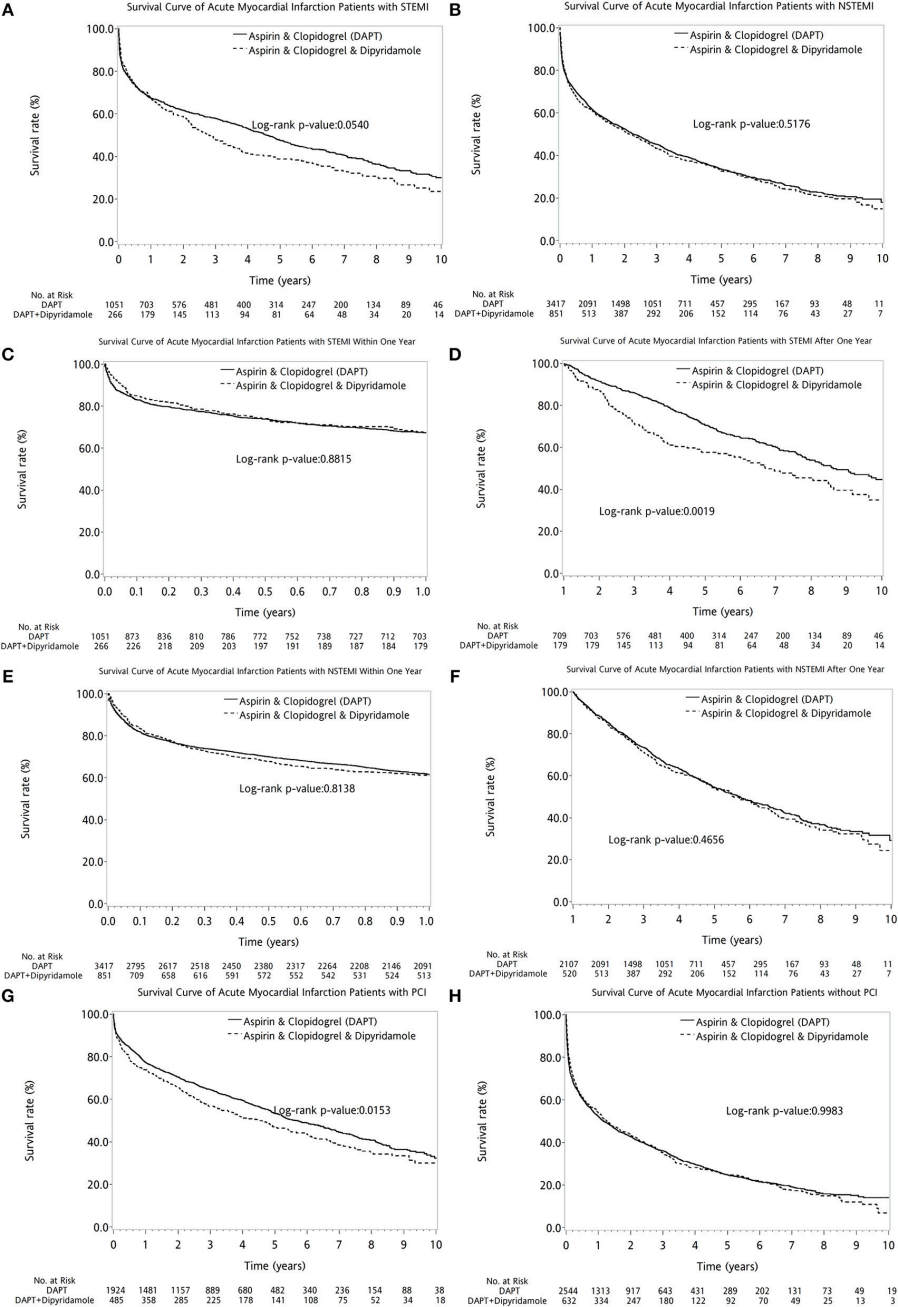
The comparison of long-term outcome between DAPT and DAPT-dipyridamole groups in patients with cerebral infarction after first AMI in AMI types and PCI subanalysis. Compared with DAPT, DAPT-dipyridamole therapy showed no extra benefit for patients after AMI, regardless of STEMI (log-rank *P* = 0.0540, **A**) or NSTEMI (log-rank *P* = 0.5176, **B**). In STEMI patients, there was no difference between DAPT and DAPT-dipyridamole subgroups within first year (log-rank *P* = 0.8815, **C**). However, DAPT subgroup revealed superior survival over DAPT-dipyridamole subgroup after first year (log-rank *P* = 0.0019, **D**) in STEMI patients. In STEMI patients, there was no significant outcome difference before (log-rank *P* = 0.8138, **E**) and after (log-rank *P* = 0.4656, Supplement 4, **F**). DAPT was superior to Dipyridamole-DAPT for patients who underwent PCI (log-rank *P* = 0.0153, **G**), but showed no survival benefit in patients who did not receive PCI (log-rank *P* = 0.9983, **H**). AMI, acute myocardial infarction; DAPT, dual antiplatelet therapy; NSTEMI, non-ST elevation myocardial infarction; PCI, percutaneous coronary intervention; STEMI, ST elevation myocardial infarction.

The two therapeutic strategies revealed no statistical differences between patients with (log-rank *P* = 0.3789, Figure 4A) and without DM (log-rank *P* = 0.1883, Figure 4B). No superiority of either therapy was detected between patients with (log-rank *P* = 0.7155, Figure 4C) or without PVD (log-rank *P* = 0.1146, Figure 4D). Compared with DAPT, DAPT-dipyridamole therapy showed no additional benefit for patients after AMI, regardless of STEMI (log-rank *P* = 0.0540, Figure 5A) or NSTEMI (log-rank *P* = 0.5176, Figure 5B). In further subanalysis of STEMI patients, there was no difference between DAPT and DAPT-dipyridamole subgroups during first year (log-rank *P* = 0.8815, Figure 5C). However, DAPT subgroup revealed superior survival over DAPT-dipyridamole subgroup after the first year (log-rank *P* = 0.0019, Figure 5D). As for outcome of NSTEMI patients, there was no significant outcome difference before (log-rank *P* = 0.8138, Figure 5E) and after (log-rank *P* = 0.4656, Figure 5F) the first year. DAPT was superior to Dipyridamole-DAPT for patients who underwent PCI (log-rank *P* = 0.0153, Figure 5G), but showed no survival benefit in patients who did not receive PCI (log-rank *P* = 0.9983, Figure 5H).

**Figure 6:**
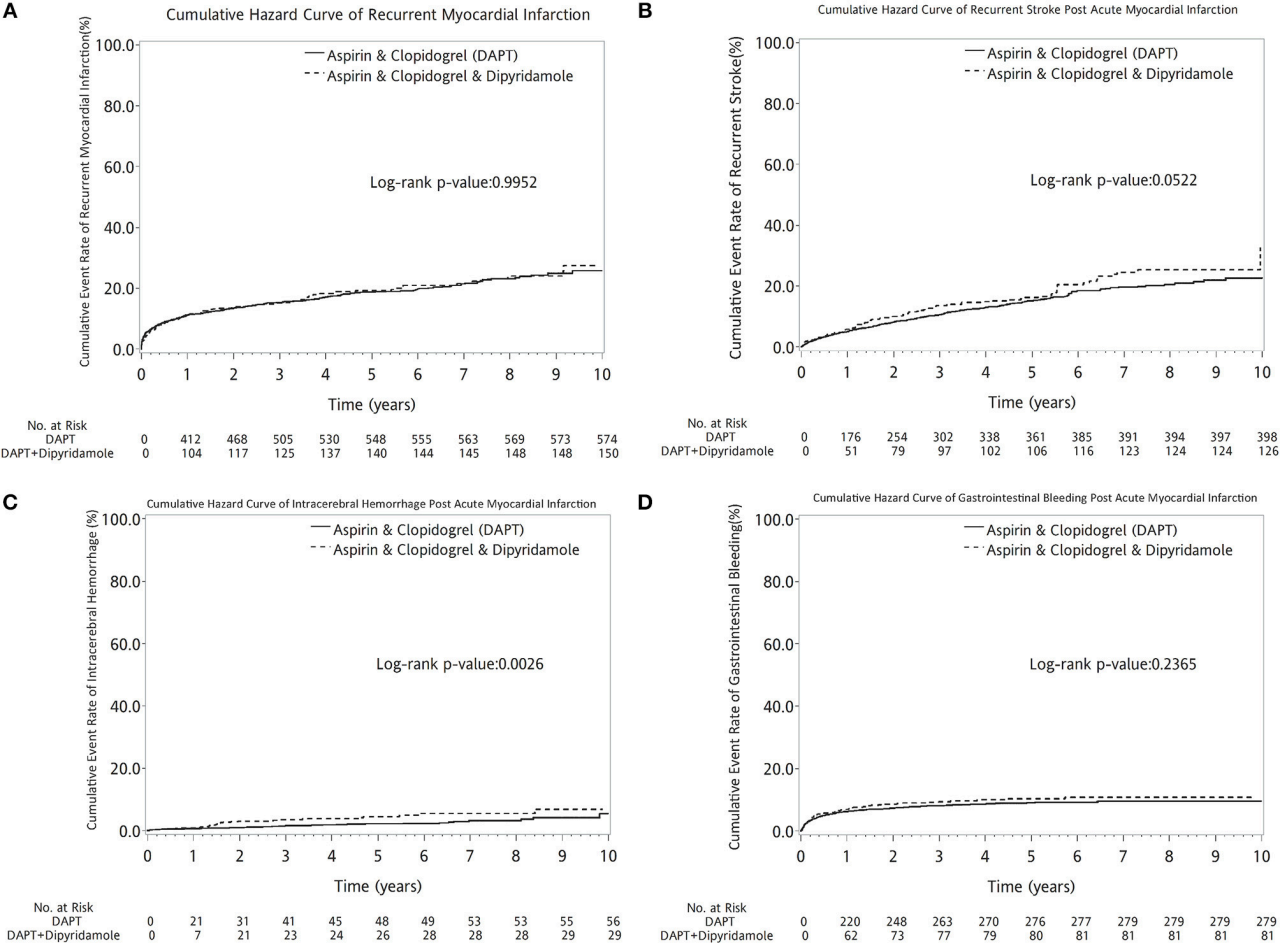
Cumulative curves of events rate in patients with previous stroke after AMI **(A–D)**. DAPT-dipyridamole did not reduce cumulative event rate of recurrent MI (log-rank *P* = 0.9952, **A**) or recurrent stroke (log-rank *P* = 0.0522, **B**) in patients after AMI. Moreover, Dipyridamole-DAPT increased the cumulative intracerebral hemorrhage rate (log-rank *P* = 0.0026, **C**), but did not affect cumulative event rate of gastrointestinal bleeding (log-rank *P* = 0.2365, **D**). AMI, acute myocardial infarction; DAPT, dual antiplatelet therapy.

DAPT-dipyridamole did not reduce cumulative event rate of recurrent MI (log-rank *P* = 0.9952, Figure 6A) or recurrent stroke (log-rank *P* = 0.0522, Figure 6B) in patients after AMI. Moreover, Dipyridamole-DAPT increased the cumulative ICH rate (log-rank *P* = 0.0026, Figure 6C), but did not affect cumulative event rate of gastrointestinal (GI) bleeding (log-rank *P* = 0.2365, Figure 6D).

Cox proportional hazard regression analysis reported mortality HRs for different variables (Table 2). Higher HR for mortality was detected for older patients (HR = 2.05; 95% CI: 1.91–2.20), patients with DM (HR = 1.36; 95% CI: 1.27–1.46), PVD (HR = 1.60; 95% CI: 1.40–1.82), ESRD (HR = 1.81; 95% CI: 1.59–2.06), and COPD (HR = 1.27; 95% CI: 1.17–1.39). In contrast, PCI was shown to reduce the risk of mortality in patients after AMI (HR = 0.51; 95% CI: 0.47–0.55). Furthermore, the use of ACEIs or ARBs (HR = 0.79; 95% CI: 0.74–0.85), statins (HR = 0.79; 95% CI: 0.71–0.88), and beta-blockers (HR = 0.82; 95% CI: 0.77–0.88) showed a significant survival benefit. Dipyridamole did not contribute to long-term survival (HR = 1.03; 95% CI: 0.95–1.12; *P* = 0.4982) in patients with stroke after first AMI.

**Table 2:** Cox proportional hazard regression on survival of patients with previous cerebrovascular accidents after first acute myocardial infarction (*N* = 5,585).

**Variables**	**HR (95% CI)**		***P*-value**
Gender (Male vs. Female)	1.01	(0.94–1.08)	0.898
Age (≧75 vs. < 75)	2.05	(1.91–2.20)	< 0.0001
Hypertension (yes vs. no)	1.06	(0.97–1.15)	0.2039
Dyslipidemia (yes vs. no)	0.98	(0.88–1.09)	0.7441
Diabetes mellitus (yes vs. no)	1.36	(1.27–1.46)	< 0.0001
Peripheral vascular disease (yes vs. no)	1.60	(1.40–1.82)	< 0.0001
End stage renal disease (yes vs. no)	1.81	(1.59–2.06)	< 0.0001
Chronic obstructive pulmonary disease (yes vs. no)	1.27	(1.17–1.39)	< 0.0001
Percutaneous coronary intervention (yes vs. no)	0.51	(0.47–0.55)	< 0.0001
ACEIs or ARBs (yes vs. no)	0.79	(0.74–0.85)	< 0.0001
Statins (yes vs. no)	0.79	(0.71–0.88)	< 0.0001
Beta blockers (yes vs. no)	0.82	(0.77–0.88)	< 0.0001
Dipyridamole (yes vs. no)	1.03	(0.95–1.12)	0.4982

*ACEI, angiotensin-converting enzyme inhibitor; ARB, angiotensin receptor blocker*.

**Figure 7:**
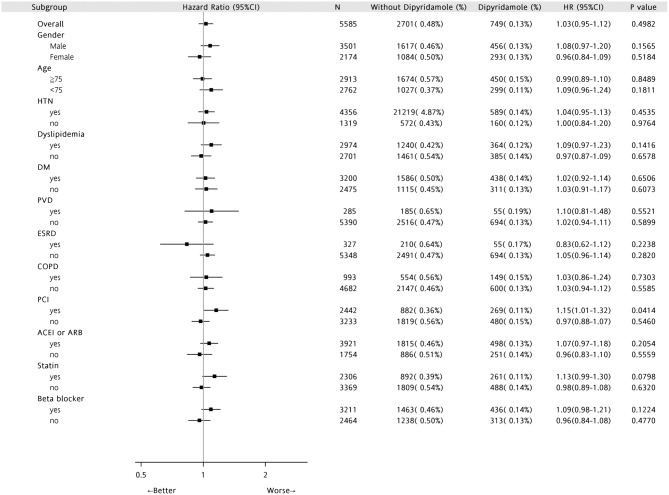
Forest plots of Hazard Ratios in patients with first AMI and previous stroke. In the overall cohort of patients, DAPT-dipyridamole therapy showed no benefit compared with DAPT, regardless of sex, age or comorbidities (HTN, DM, PVD, ESRD, COPD) or drugs. Nevertheless, DAPT has better survival than DAPT-dipyridamole in patients underwent PCI (*P* = 0.0414). AMI, acute myocardial infarction; DAPT, dual antiplatelet therapy; PCI, percutaneous coronary intervention.

Forest plots of hazard ratios (Figure 7) indicated that in the overall cohort of patients, DAPT-dipyridamole therapy showed no benefit compared with DAPT, regardless of sex, age, or comorbidities (HTN, DM, PVD, ESRD, COPD) or drugs. Nevertheless, DAPT has better survival than DAPT-dipyridamole in patients underwent PCI (*P* = 0.0414).

## Discussion

This nationwide study is the first study to evaluate the impact of dipyridamole added to DAPT on secondary stroke prevention and long-term outcomes in patients with cerebral infarction after first AMI. It showed that DAPT-dipyridamole did not improve long-term survival in patients with AMI and previous stroke. Moreover, Dipyridamole-DAPT did not reduce the cumulative event rate of recurrent MI or stroke in patients with stroke after AMI, but increased cumulative ICH rate, without impacting the incidence of GI bleeding.

### Dipyridamole-DAPT had similar first-year survival and worse outcome after first year than DAPT in patients with previous stroke after AMI

TAPT comprising cilostazol decreased MACE risks in patients with AMI, which was proved in several large observational clinical trials and meta-analyses (11, 26–29), but the results need further randomized trials for confirmation. Cilostazol reduced platelet aggregation, induced vascular dilatation, and limited intimal hyperplasia, driving its extensive application in patients after stenting (13, 30). Dipyridamole has a similar mechanism of action to that of cilostazol, and is the first-line drug for no-reflow phenomenon during primary PCI (31). Nevertheless, among stented patients with high platelet reactivity, adding dipyridamole to DAPT does not reduce platelet reactivity (32), and any additional clinical benefit provided by dipyridamole may be attributed to other mechanisms of action (33). For patients with concomitant AMI and old stroke, no previous study addressed the long-term outcomes after AMI of Dipyridamole-DAPT, and our study did not reveal its superiority compared with DAPT and Dipyridamole-DAPT had even worse outcome after first year. Previous trial proved that there was greatest incremental risk of bleeding of DAPT in the first year (34). Recent *post hoc* analyses also reported that DAPT had similar bleeding risk compared to monotherapy thereafter (35). In this study, DAPT-dipyridamole subgroup presented incremental cumulative event rate of ICH after the first year in the cumulative hazard curve, with a crossed over linear trend in the beginning and dividing apart incrementally after 1 year. Aforementioned studies supported our result that comparing to stationary bleeding risk of DAPT after 1 year, additional ICH incidence of Dipyridamole-DAPT in patients diminished its antithrombotic benefit after the first year.

### Dipyridamole-DAPT was related to significantly lower survival after 1 year in younger patients with previous stroke after AMI

Enhanced permeability of the vascular wall after prolonged ischemia and hypoxia greatly increases the chances of hemorrhagic transformation after the release of the oedema (33). Hemorrhagic transformation is a frequent spontaneous complication of ischemic stroke (33). Dipyridamole use was associated with an increased risk of subarachnoid hemorrhage, and half of all subarachnoid hemorrhages occurred in patients younger than 55 years old (34, 35). Previous studies supported the results of this study that Dipyridamole-DAPT had significant lower survival after 1 year in younger patients with previous stroke after AMI, which might be explained by increase of intracranial bleeding in Dipyridamole-DAPT group.

### Dipyridamole-DAPT had similar outcome as DAPT in patients with DM and previous stroke after first AMI

Adding cilostazol to the routine DAPT was reportedly associated with good results in patients with DM and AMI, in whom the risk of thrombotic events development after stenting implantation is high (9, 36). Even though the mechanism of action of dipyridamole is similar to that of cilostazol, no previous study assessed the efficacy of Dipyridamole-DAPT in patients with DM and both stroke and AMI. Antiplatelets therapy was found to reduce prothrombotic environment in DM, but with a higher general risk of bleeding, specifically of intracranial hemorrhage (37). Previous studies supported that Dipyridamole-DAPT did not have additional benefit than DAPT in patients with DM and previous stroke after AMI. The superiority of DAPT-dipyridamole therapy might be impaired by increase of intracranial bleeding.

### Dipyridamole-DAPT did not have additional benefit on PCI than DAPT in patients with previous stroke after AMI

In patients with high post-treatment platelet reactivity despite conventional DAPT, cilostazol intensifies platelet inhibition (13). Cilostazol-based TAPT proved superiority in high-risk patients after stent deployment (9, 38), but the benefit was not proved in other studies (13, 39). Moreover, cilostazol-based TAPT achieved lower post-treatment platelet reactivity, without a reduction of ischemic events after PCI (13). Enhanced platelet inhibition did not decrease the actual number of clinical events (13). These facts support the results of the present study that Dipyridamole-DAPT after AMI does not add additional benefit compared with DAPT for patients who underwent PCI.

### Dipyridamole-DAPT increased the incidence of ICH and had no better effect on secondary stroke prevention than DAPT in patients with stroke after AMI

Dipyridamole is synergistic with aspirin for secondary stroke prevention (40), but no previous study investigating the impact of Dipyridamole-DAPT on secondary stroke prevention compared with DAPT for patients with AMI and previous stroke is currently available. Dipyridamole has non-platelet-related effects that can reduce plasma levels of von Willebrand factor. Therefore, Dipyridamole-DAPT showed no benefit in recurrent stroke prevention because of the significantly increased risk of major bleeding (19, 20, 41, 42). A previous randomized clinical trial reported an increased bleeding rate in patients under long-term Dipyridamole-DAPT and a trend to increase treatment discontinuation (19). The overlapped risk factors of bleeding and ischaemic events lead to an increased rate of thrombotic events due to the progressive recovery of platelet function and coagulation activity after discontinuation of antithrombotic drugs (43). Therefore, additional benefit of Dipyridamole-DAPT for recurrent stroke prevention was masked by the concomitant risk of thrombosis in addition to bleeding events, which support the results of the present study that Dipyridamole-DAPT has no benefit for secondary stroke prevention, but increases the incidence of ICH in patients with stroke after AMI.

## Study limitations

There were several limitations in our study. First, the stroke was not identified by medical record, but was defined by ICD-9-CM code. We defined previous stroke with ICD-9-CM code (ICD: 433–438, A292–294) after excluding hemorrhagic stroke (ICD-9 code 430–432; A290–A291). Previous study showed that the sensitivity of ICD code 430–438 for any cerebrovascular disease achieving was ≥82%. The specificity and negative predictive value were both ≥95%, and positive predictive value of these codes for any cerebrovascular disease was ≥81% (44). Secondly, etiology, severity, functional status, and outcome of previous stroke, such as the scores of modified Rankin Scale and the National Institutes of Health Stroke Scale, could not be assessed completely in this study. Thirdly, the outcomes were not adjusted using antihypertensive treatment. However, a 1:4 propensity score matching ratio was adopted based on multiple variables in this study. Furthermore, there were no difference of percentage of hypertension and antihypertensive medication, including ACEI or ARBs and beta-blockers between Dipyridamole-DAPT and DAPT group. Furthermore, the indices to evaluate the severity of comorbidities and stroke-related risk factors, such as body mass index, glycated hemoglobin level, blood pressure, ankle-brachial index, left ventricular ejection fraction, functional status before and after AMI were unavailable in the database. Moreover, the results of standard laboratory tests evaluating renal, liver, and coagulation functions were not available; this fact might interfere with drug-drug interaction and bleeding risk. Finally, this was a nationwide, case-control study, and future prospective randomized studies are required to confirm obtained findings.

## Strengths of this study

This is the first study to investigate the impact of dipyridamole added on DAPT on stroke prevention and long-term outcomes in patients with previous stroke after first AMI. The large database and propensity score reduced the variability in sampling statistics. This study offers clinically relevant information on antiplatelet strategies for medical practitioners.

## Conclusions

This nationwide study showed that Dipyridamole-DAPT did not improve long-term survival in patients with stroke after AMI, and was related to poor outcomes after 1 year. Furthermore, Dipyridamole-DAPT had significant worse outcome than DAPT in patients received PCI, younger and STEMI patients after first year. Dipyridamole-DAPT did not reduce cumulative event rate of recurrent MI or recurrent stroke in patients after AMI, but increased cumulative ICH rate. Cox analysis revealed that Dipyridamole-DAPT has not shown benefit for long-term survival in patients with previous stroke after AMI.

## Availability of data and materials

Data are available from the National Health Insurance Research Database (NHIRD) published by Taiwan National Health Insurance (NHI) Bureau. Due to legal restrictions imposed by the government of Taiwan in relation to the “Personal Information Protection Act,” data cannot be made publicly available. Requests for data access can be sent as a formal proposal to the NHIRD (http://nhird.nhri.org.tw).

## Ethics statement

The Institutional Review Board (IRB) of the Kaohsiung Veterans General Hospital approved this study (No. VGHKS14-CT7-07). Written informed consent was not required for this study as National health insurance research database consists of de-identified secondary data for research purposes, and the IRB of Kaohsiung Veterans General Hospital issued a formal written waiver of the requirement for informed consent.

## Author contributions

All authors listed have made a substantial, direct and intellectual contribution to the work, and approved it for publication.

### Conflict of interest statement

The authors declare that the research was conducted in the absence of any commercial or financial relationships that could be construed as a potential conflict of interest.
